# Effect of bitter orange blossom distillate on anxiety and sleep disorder in mothers with infants admitted to neonatal intensive care unit: A Randomized controlled clinical trial

**DOI:** 10.1371/journal.pone.0306887

**Published:** 2024-08-12

**Authors:** Zahra Dehghan, Seyedeh Roghaye Jafarian Amiri, Seyyed Ali Mozaffarpur, Hoda Shirafkan, Afsaneh Arzani

**Affiliations:** 1 Student Research Committee, Babol University of Medical Sciences, Babol, Iran; 2 Nursing Care Research Center, Health Research Institute, Babol University of Medical Sciences, Babol, Iran; 3 Traditional Medicine and History of Medical Sciences Research Center, Health Research Institute, Babol University of Medical Sciences, Babol, Iran; 4 Social Determinants of Health Research Center, Health Research Institute, Babol University of Medical Sciences, Babol, Iran; 5 Non-Communicable Pediatric Diseases Research Center, Health Research Institute, Babol University of Medical Sciences, Babol, Iran; University of Milan, ITALY

## Abstract

**Objective:**

Mothers of neonates admitted to the intensive care unit are prone to sleep disorders due to stress and anxiety. Some herbs have anti-anxiety and sedative properties. This study aimed to investigate the effect of bitter orange blossom distillate on anxiety and sleep disorders in mothers with infants admitted to the neonatal intensive care unit.

**Methods:**

This randomized controlled clinical trial was conducted from 2021 May 15 to 2022 February 2, on 60 mothers with NICU-admitted infants in one of the medical teaching centers affiliated with Babol University of Medical Sciences, Iran. Permuted block randomization was used to assign the mothers to the experimental (bitter orange blossom distillate syrup) and control groups (syrup prepared from water and sugar). Given the impossibility of blinding the participants, allocation concealment was used to prevent the researcher from predicting the future allocation of the samples to the groups. In addition, the statistical consultant received coded data pertaining to the type of intervention in each group. A demographic questionnaire, the Spielberger State and Trait Anxiety Inventory (STAI), and the General Sleep Disorder Scale were employed to collect data. We assessed the level of anxiety and sleep disorder (in both groups before the intervention and again after the last day of the intervention). Data were analyzed using SPSS V22 software. A P-value less than 0.05 were considered significant.

**Results:**

The mean and standard deviation of the sleep disorder score in the intervention group from (50.26±5.81) to (44.70±6.94) and in the control group from (50.46±6.95) to (48.53±8.62) changed. The covariance test showed that bitter orange blossom distillate syrup has a significant effect on the improvement of sleep disorders (P = 0.01, Effect size = 0.09), but there was no significant difference in the State and Trait anxiety level of mothers (P = 0.122, 0.144 and Effect size = 0.04, 0.03) respectively.

**Conclusions:**

Due to the positive effect of bitter orange blossom distillate syrup on sleep disorders of mothers with hospitalized babies, this low-cost and low-risk intervention is recommended.

**Trial registration:**

This study is registered at clinicaltrials.gov as Trial ID = IRCT20201209049666N1.

## Introduction

Over 10–15% of the world’s live born infants are admitted to a neonatal intensive care unit (NICU) annually [1]. The parents of infants admitted to the NICU experience anxiety, depression, stress, and sleep disorders [2]. Maternal anxiety and separation from the infant inhibit oxytocin secretion and reduce breastfeeding, resulting in diminished loving and responsible maternal behaviors and an increasingly higher chance of developing postpartum depression [3, 4].

Stress and anxiety make these mothers susceptible to sleep disorders [5]. Sleep is an organized behavior repeated every day as a vital necessity that is based on biological rhythm. Adequate sleep is essential for a mother’s psychological functioning and ability to support and participate in the care of her infant [6].

Taking hypnotic drugs is one way of improving sleep disorders. Nonetheless, as the majority of hypnotic drugs are contraindicated in the postpartum period because they cross into mother’s milk, non-pharmacological methods without side effects should be utilized as a safe alternative for treating sleep problems in the postpartum period [7].

In this regard, various caregiving methods are available, including complementary medicine. Complementary medicine is a set of holistic diagnostic, therapeutic, or preventive practices that are used worldwide in conjunction with conventional medicine [8]. As a form of complementary and alternative medicine, medicinal plants are widely and extensively used throughout the world [9]. According to a report from the World Health Organization, the use of herbal medicines is on the rise in most countries due to their efficacy, lower risk and cost, and greater availability [10].

Bitter orange (*Citrus aurantium*) blossoms are one of the most widely used native medicinal plants in Iran. The flowers of this plant are used to treat nervous disorders such as hysteria, convulsion, and neurasthenia in traditional Iranian medicine. This plant is also recognized as a sedative, soporific agent, appetizer, and reliever of heart palpitations. According to research, bitter orange blossoms contain compounds such as linalool, linalyl acetate, limonene, coumarin, and flavonoids. The contents of these compounds are greater in the blossoms than in the leaves [11].

Abbasnia et al. conducted an experiment in which they tested the effect of bitter orange blossom extract on anxiety and sleep in 80 laboratory mice. They reported that the extract prolonged sleep duration and reduced anxiety in the mice [12].

Few studies have been conducted in humans on the effects of bitter orange blossom distillate (BOBD) in the postpartum period, and most of them have been aromatherapy or laboratory studies. Sharifipour et al. concluded in the study that the aroma of bitter orange blossom distillate reduced anxiety following cesarean section [13]. In another study, Akhlaghi et al. demonstrated that BOBD could be used as an effective preoperative anxiolytic prodrug [14].

There is a high incidence of postpartum anxiety and sleep disorders among mothers with hospitalized infants [5], which can affect both mothers and infants. In addition, many mothers believe that herbal products are superior to chemical medications and prefer using them. Compounds found in the bitter orange are known to have soothing effects. In light of the aforementioned considerations and due to the lack of research in this area, this study was designed to determine the effect of BOBD on postpartum anxiety and sleep disorders in mothers with NICU-admitted infants.

## Materials and methods

The study protocol was granted ethical approval by the Ethics Committee of Babol University of Medical Sciences (IR.MUBABOL.REC.2019.393) and we confirm that all methods were performed in accordance with the relevant guidelines and regulations set by declaration of Helsinki. Written informed consent/informed assent was obtained from all mothers who participate in the study. Also we registered in the Iranian Registry of Clinical Trials (IRCT20201209049666N1).

Subsequently, a randomized controlled clinical trial was conducted in 2021 May 15 until 2022 February 2, on 60 mothers with NICU-admitted infants in one of the medical teaching centers affiliated to Babol University of Medical Sciences, Iran. The primary outcomes in this study was to investigate the effect of bitter orange blossom distillate on anxiety and sleep disorder in mothers with infants admitted to NICU.

After coordinating with the related authorities, the researcher visited the NICU to be introduced to the mothers (in the maternity ward and for the objectives of the research to be explained to them. Those who scored 43 or higher on the General Sleep Disturbance Scale (GSDS) were recruited as the eligible participants. Permuted block randomization was used to assign the mothers to the experimental and control groups, with blocks of 4 consisting of two A’s and two B’s created in a 1:1 ratio. In each block, the order of receiving A and B was chosen at random from a variety of permutations (two A’s and two B’s). Group A was the experimental group and group B the control group. Given the impossibility of blinding the participants, allocation concealment was used to prevent the researcher from predicting the future allocation of the samples to the groups. In addition, the statistical consultant received coded data pertaining to the type of intervention in each group. (While blinding requires similarity, it was not possible to comply with this principle in the case of Bitter Orange Blossom Distillate because its smell has therapeutic effect which is part of the study. We didn’t add this smell to the control group. The smell of syrup was different in the two groups, therefore, the patients were not blinded, but the shape, color, and consistency were the same. The study was conducted during the COVID-19 pandemic, and there was no possibility of contamination between mothers, as mothers consumed syrup at home).

The sample size was obtained using G*Power 3.1.9.2 software and considering the effect size of 0.3 [13], the error level of 5%, and the power of 80%, equal to 24 mothers in each group. Considering the dropout rate of 20%, the sample size was 30 mothers in each group. A total of 60 samples were included in the study.

**Fig 1:**
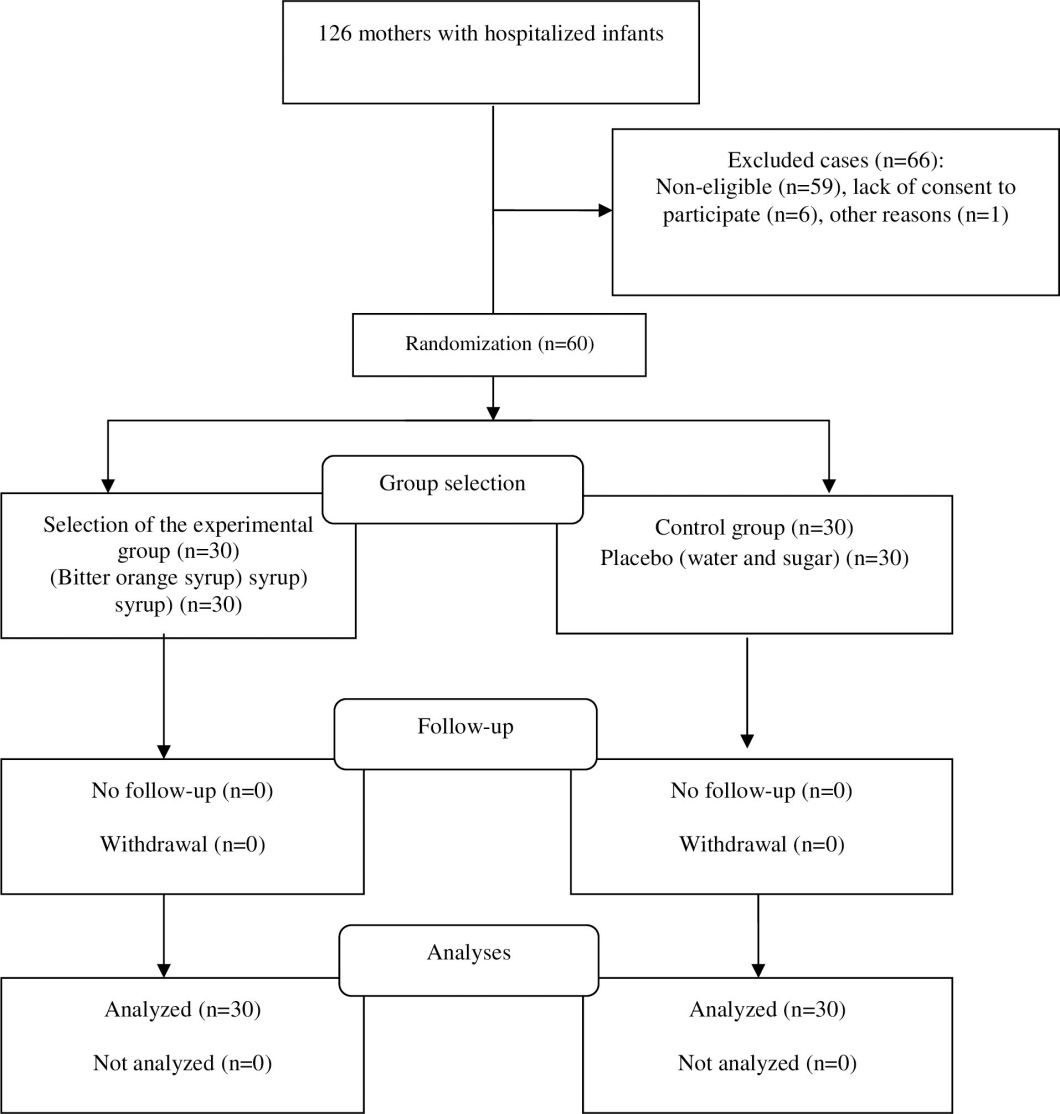
Consort algorithm for selection of the participants.

A flow diagram describing mother selection is shown in Fig 1. The inclusion criteria comprised being literate, not having a history of neurological and mental illnesses, the infants being in the NICU for seven days, the mothers scoring 43 or higher on the GSDS, not having allergies to medicinal and food plants, not having a history of diabetes, not having had a premature or NICU-admitted infant before, and not having pregnancy complications such as diabetes, eclampsia, and heart problems. The exclusion criteria were the infant’s hospitalization in the NICU for fewer than 14 days, the mother’s refusal to continue participation in the study, not consuming syrup for a day for any reason, the occurrence of unpleasant events for the mother, the mother’s sensitivity to BOBD, and the mother’s need for sleep disorder or anxiety medications during the intervention.

The eligible participants signed a written consent form to enter the research. A demographic questionnaire, the Spielberger State and Trait Anxiety Inventory (STAI), and the GSDS were employed to collect data. Numerous studies have investigated and confirmed the validity and reliability of the STAI [15, 16].

STAI: This instrument includes separate self-assessment subscales to measure state and trait anxiety. The state anxiety scale consists of 20 items that evaluate an individual’s feelings in "this moment and at the time of response". The trait anxiety scale also includes 20 statements that measure people’s general and ordinary feelings.

Depending on the response, each statement on the STAI is assigned a weight between 1 and 4. A score of 4 indicates a high level of anxiety. The 10 state anxiety scale statements and the 11 trait anxiety scale statements are scored in this way. For scoring the other items (which include 10 statements measuring state anxiety and nine statements assessing trait anxiety) a high rating for each item indicates absence of anxiety.

When it was taken into account that scoring was reversed for some statements, the total score of the twenty statements in each scale was calculated in order to obtain the anxiety score for each participant in each of the two scales. Consequently, the scores for each participant in both scales were in the range of 20–80 [17].

The following are the interpretations of trait and state anxiety scores based on norming research for trait and state anxiety tests. The total score for both subscales ranged from 20 to 80, with scores between 20 and 40 indicating mild anxiety, 41–60 denoting moderate anxiety, and 61–80 representing severe anxiety [16].

This tool’s validity was confirmed for application in Iran [18]. Reliability level for state and trait anxiety, as indicated by Cronbach’s alpha coefficient, was consistently reported in most studies ranged from 0.74 to 0.90 [16, 18, 19] and for our study was 0.79. The GSDS, which consists of 21 items and evaluates the frequency of sleep disturbances over the previous week, was used to measure sleep disorders. The items are scored on an eight-point Likert scale, ranging from 0 (never) to 7 (every day), and completion of the scale takes less than 10 minutes. These items measure sleep quality, sleep onset latency, sleep quantity, sleep maintenance, early awakening, use of medications to sleep better, drowsiness, and the effect of drowsiness on daily functioning. The total score for GSDS ranges from 0 to 147, with higher scores indicating more severe sleep disorders. The cut-off scores of 43 and 3 distinguish good sleep from poor sleep for the entire scale and for each sleep domain. Based on DSM-IV criteria for primary insomnia, a mean score of 3 or higher indicates that sleep was disrupted in the previous week for three nights or more [20].

Validation of the general sleep disorder scale among Chinese American parents with a hospitalized infant has been done in Lee’s (2007) study. Cronbach’s alpha coefficients for the English and Chinese versions were 0.85 and 0.81, respectively [21]. A literature review indicates that the GSDS has not yet been implemented in Iran. In light of this, this study investigated its validity and reliability. Ten experts, including professors, nurses, and mothers, were surveyed to determine the content and face validity of the scale. The content validity index (CVI) and content validity ratio (CVR) values were 0.62 and 0.79, respectively, and Cronbach’s alpha reliability coefficient for this scale with 20 participants was 0.87.

The participants in the experimental group (n = 30) took 100 mL syrup prepared by the researcher (under the supervision of an expert group in Iranian traditional medicine) three times daily at 8 AM, 2 PM and 8 PM for seven days. (The syrup was prepared as follows: The researcher gradually dissolved sugar (66.7 g) in 100 mL BOBD until a uniform solution was obtained (sugar to distillate ratio is based on the USP (United States Pharmacopeia # 1) standard). Afterward, 30 mL of the resulting solution was further diluted with 70 mL water to raise the volume to 100 mL).

In the control group (n = 30), the participants were given 100 mL syrup with the same percentage of the sweetness of bitter orange syrup three times daily at the same times as the experimental group for seven days. The researcher conducted daily telephone follow-ups to ensure that all the participants in the groups consumed the prepared solutions. It should be mentioned that BOBD was prepared for all mothers from the same company and in the same way.

The STAI and GSDS were completed at baseline and post-intervention (Eighth day) by the mothers in the two groups.

### Statistical analysis

The collected data were analyzed in SPSS Version 22. Descriptive statistics were presented as mean (± standard deviation (SD)), frequency, and percentage. To assess the effect of intervention on primary outcomes we used analysis of covariance, after verifying and confirming the assumptions of ANCOVA—specifically, the normality within subgroups and the absence of significant interaction between group and covariate—the p-value for the group effect was reported. The level of significance was set at P <0.05. In all tests, a two-tailed statistical test was utilized.

## Results

In this study, 60 mothers with NICU-admitted infants were studied. The mothers in the two groups were homogenous in terms of demographic variables (education, occupation, insurance coverage, type of delivery, and certain infant characteristics, such as gestational age, birth weight, gender, and birth order rank) (Table 1).

**Table 1:** Baseline data of participants.

Variables		Total Frequency (percent)	Experimental group Frequency (percent)	Control Group Frequency (percent)
Education	Without high school diploma	8 (13.3)	1 (3.3)	7 (23.3)
	High school diploma	28 (46.7)	16 (53.3)	12 (40.0)
	Bachelor’s degree or higher	24 (40)	13 (43.4)	11 (36.7)
Occupation	Employee	10 (16.7)	5 (16.7)	5 (16.7)
	Self-employed	50 (83.3)	25 (83.3)	25 (83.3)
Insurance coverage	Yes	56 (93.3)	29 (96.7)	27 (90.0)
	No	4 (6.7)	1 (3.3)	3 (10.0)
Type of delivery	Natural	7 (11.7)	3 (10.0)	4 (13.3)
	C-section	53 (88.3)	27 (90.0)	26 (86.7)
Birth rank	First child	27 (45.0)	14 (46.7)	13 (43.3)
	Second child, third child, etc.	33 (55.0)	16 (53.3)	17 (56.7)
Gender of the newborn	Female newborn	31 (51.7)	16 (53.3)	15 (50.0)
	Male newborn	29 (48.3)	14 (46.7)	15 (50.0)
Birth weight (< 5 pounds, 8 ounces)	Extremely low birth weight babies (ELBW)	9 (15.0)	4 (13.3)	5 (16.7)
	Very low birth weight (VLBW)	11 (18.3)	7 (23.3)	4 (13.3)
	Low birth weight (LBW)	30 (50.0)	15 (50.0)	15 (50.0)
	Normal weight	10 (16.7)	4 (13.3)	6 (20.0)
Gestational age	Premature	52 (86.7)	26 (86.7)	26 (86.6)
	Mature	8 (13.3)	4 (13.3)	4 (13.3)

The mean sleep disorder scores of the control and experimental groups did not differ at baseline (Table 2). It was discovered that bitter orange syrup significantly improved sleep disorders (as sleep disorder was improved by 5.56 units in the experimental group but only 1.93 units in the control group). In addition, based on the effect size of 0.09, the intervention had a moderate impact on improving sleep disorder in the experimental group (P = 0.011, effect size = 0.09). In this study, effect sizes < 0.009, 0.01–0.059, 0.06–0.13, and > 0.13 were interpreted as having no effect, a slight effect, a moderate effect, and a considerable effect, respectively [22].

**Table 2:** Comparison of sleep disorder and trait and state anxiety between the experimental and control groups.

Dependent Variable		Experimental group Mean ± standard deviation	Control Group Mean ± standard deviation	P value^*^	Effect size (partial eta square)
Sleep disorder	Baseline	50.26±5.81	50.46±6.95	0.011	0.09
	The seventh day after the intervention	44.70±6.94	48.53±8.62		
State anxiety	Baseline	41.76±8.19	44.53±12.21	0.122	0.04
	The seventh day after the intervention	38.86±7.20	43.10±11.91		
Trait anxiety	Baseline	39.73±7.50	42.16±10.28	0.144	0.03
	The seventh day after the intervention	38.50±6.17	41.90±9.92		

* Analysis of Covariance: In the analysis of covariance (ANCOVA), the covariates were the baseline scores for sleep disorder, state anxiety, and trait anxiety (before the intervention). The dependent variables were the post-intervention scores for sleep disorder, state anxiety, and trait anxiety. The fixed factors were the intervention and comparison groups, and the p-values were used to evaluate the effect of the groups

Also, based on the results of the t-test, there was no statistically difference between the control and experimental groups in the mean baseline scores of state and trait anxiety. Likewise, there was no statistically significant difference between the control and experimental groups in mean post-intervention scores for state and trait anxiety (P = 0.122 and 0.144) respectively. While state and trait anxiety scores changed in the experimental group, this difference was not significant. In other words, the effect of bitter orange syrup on anxiety in mothers was statistically non-significant.

Table 3 compares the sleep disorders of the two groups of mothers based on some variables such as birth rank, gestational age, and infant weight. As Table 3 reveals, bitter orange syrup did not contribute to mothers’ sleep disorder in the participants based on birth rank for first and second child (P = 0.043 and 0.034) respectively.

**Table 3:** Comparison of sleep disorder before and after the intervention based on birth rank, birth weight, and gestational age in the experimental and control groups.

Variable		Group	Sleep disorder before the intervention	Sleep disorder after the intervention	P value*	Effect size partial eta square
Birth rank	First child	Experimental	50.00 ± 6.28	46.14±7.55	0.043	0.15
		Control	48.46±4.46	47.46±8.30		
	Second child, etc.	Experimental	50.50±5.57	43.44±6.33	0.034	0.14
		Control	52.00±8.18	49.35±9.03		
Low birth weight (<5 pounds, 8 ounces)	Extremely low birth weight infant (< 1000 g)	Experimental	45.00±1.41	35.50±4.79	0.079	0.43
		Control	47.20±2.28	48.60±6.98		
	Very low birth weight (1000–1499 g)	Experimental	49.14±4.22	47.14±3.97	0.359	0.11
		Control	45.50±1.73	40.75±8.30		
	Low birth weight (1500–2499 g)	Experimental	50.86±5.69	46.40±6.97	0.227	0.05
		Control	49.26±4.65	46.73±5.75		
	Normal weight (> 2500 g)	Experimental	55.25±7.93	43.25±6.34	0.045	0.46
		Control	59.9±5.00	58.16±9.43		
Gestational age	Premature < 34 weeks	Experimental	49.90±5.59	45.09±7.85	0.187	0.05
		Control	48.84±4.35	46.21±7.28		
	Late prematurity 34–36 weeks	Experimental	47.80±2.86	44.20±2.77	0.042	0.36
		Control	50.14±9.46	50.28±9.63		
	Mature > 37 weeks	Experimental	55.25±7.93	43.25±6.34	0.103	0.44
		Control	58.75±8.14	56.50±9.43		

^*^ Analysis of Covariance: The data is categorized by birth rank, low birth weight, and gestational age. For each category, an analysis of covariance (ANCOVA) was conducted. In the ANCOVA, the baseline sleep disorder scores (before the intervention) served as the covariate, while the post-intervention sleep disorder scores were the dependent variable. The group was considered the fixed factor, and the p-values were used to evaluate the effect of the groups.

Regarding birth weight, bitter orange syrup caused a significant difference between the two groups in terms of sleep disorders improvement before and after the intervention in mothers of babies with normal birth weight (P = 0.045; effect size = 0.46). However, it did not affect sleep disorders in the birth weight subgroups except for infant with normal birth weight. Concerning gestational age, the bitter orange syrup significantly improved sleep disorder only in mothers with late preterm babies (34–36 weeks of gestation; P = 0.042).

## Discussion

The results revealed that bitter orange syrup improved sleep disorders in mothers with NICU-admitted infants, but there was no significant difference in state or trait anxiety levels between the experimental and control groups. According to the findings in the study by Abbaspoor et al., aromatherapy with bitter orange improved the quality and duration of sleep in postmenopausal women. These findings are consistent with those of the present study, despite the fact that the target population in their study comprised postmenopausal women and that in our study was reproductive age mothers with newborns in the NICU [23].

In this regard, the findings of the research by Arab Firouzjaei indicated that aromatherapy with certain medicinal plants, such as bitter orange, could improve sleep quality in elderly individuals with heart failure [24]. Although their study population consisted of older adults with heart failure or of patients undergoing coronary artery interventions, Asghari et al. found that the bitter orange aroma improves sleep quality in patients undergoing percutaneous coronary interventions [25].

In line with our findings, Gharaee et al. found that aromatherapy with sweet orange and bitter orange extracts affected sleep quality and quantity in patients with the acute coronary syndrome [26]. The aforementioned studies confirmed the effect of bitter orange on human participants, except that they all evaluated the effect of aromatherapy on sleep quality and quantity. In fact, a review of the literature revealed that no study investigated the effect of bitter orange syrup on pregnant women with NICU-hospitalized infants, but their results were interpreted because they were similar to the present research.

In our study, bitter orange syrup did not cause a significant difference between the participants in state and trait anxiety; however, it reduced state anxiety by 2.91 units and trait anxiety by 1.23 units. The study by Moslemi et al. revealed that the aroma of bitter orange blossom effectively reduced anxiety in patients with acute coronary syndromes (inhalation of bitter orange blossom aroma had anti-anxiety effects) [27].

The study conducted by Abbasnia et al. also indicated that the aqueous extract of bitter orange blossom induced sleep and reduced anxiety in laboratory mice [12]. Even though this study was conducted on animal models, its results support our findings. In our study, state and trait anxiety levels improved in the experimental group relative to the control group, although the improvement was not statistically significant.

In support of our findings, Abdollahi et al. found that aromatherapy with bitter orange blossom significantly reduced dizziness and anxiety in diabetic patients. In other words, aromatherapy with bitter orange extract is a potentially effective intervention for reducing anxiety and fatigue in diabetic patients [28].

Another study found that aromatherapy with bitter orange was effective in reducing anxiety during the first stage of labor [29]. In addition, the results of our study demonstrated that bitter orange syrup could reduce the anxiety score of mothers whose infants were hospitalized in the NICU.

State and trait anxiety scores were not significantly different between the groups in our study. It can be argued that the small the sample size explains the lack of significance, and it is possible that as the sample size increases, the difference in state and trait anxiety scores between the experimental and control groups becomes statistically significant.

As mentioned above, no studies were found on the effect of bitter orange syrup on sleep disorders and anxiety in humans in the literature review. Few studies have reported the effect of bitter orange blossom aroma on human anxiety, with the majority of studies focusing on animal models. In order to interpret the results in this regard, there was no relevant research available about the effect of bitter orange syrup in improving mothers’ sleep disorders in mothers in relation to variables such as birth order rank, gestational age and birth weight to interpret the results in this section.

In addition, this trial had a small sample size. Although the intervention with bitter orange blossom distillate led to a statistically significant difference between the two groups of mothers in sleep disorder (a moderate effect based on size effect), it exhibited no statistically significant effect on the mothers’ anxiety scores. Therefore, it is advised to conduct more extensive studies with larger samples. In addition, our limitation in this study was conducted during the COVID-19 pandemic, which restricted sample availability. Lastly, the impossibility of blinding the samples (due to the smell of Bitter Orange Blossom Distillate) and outcome assessor (the outcome was evaluated subjectively and self-reported by the mothers), but the researcher and the data analyst were blind.

## Conclusion

This study provided evidence concerning the efficacy of bitter orange syrup in treating sleep disorders in mothers whose infants were hospitalized in the NICU. Consequently, this syrup can be used as a simple, inexpensive, efficient, and effective intervention.

## Supporting information

S1 Checklist CONSORT checklist. (DOCX)

S1 File Study protocol. (DOCX)

## Abbreviations

BOBD: Bitter Orange Blossom Distillate
NICU: Neonatal Intensive Care Unit
GSDS: General Sleep Disturbance Scale
STAI: State and Trait Anxiety Inventory
CVI: Content Validity Index
CVR: Content Validity Ratio
