# Supplementary material for: Effect of bitter orange blossom distillate on anxiety and sleep disorder in mothers with infants admitted to neonatal intensive care unit: A Randomized controlled clinical trial
Source: PLoS One. 2024 Aug 12;19(8):e0306887. doi: 10.1371/journal.pone.0306887 (PMC11318878; doi:10.1371/journal.pone.0306887)
Supplement: S1 File — (DOCX) [file pone.0306887.s002.docx]

**Research protocol**

**Project title**

Effect of Bitter Orange Blossom Distillate on Anxiety and Sleep Disorder in Mothers with Infants Admitted to Neonatal Intensive Care unit: A Randomized Controlled Clinical Trial

**Protocol summary**

**Study aim**

The Effect of Citrus Aurantium distillate on Anxiety and Sleep Disorder in mothers with neonate admitted to neonatal intensive care unit.

**Design**

The clinical trial will have a control group and an intervention group, a randomized controlled trial, a method of randomization of permutation blocks and a sample size of 60 people.

**Settings and conduct**

This study is a two-group clinical trial performed on mothers with infants admitted to the NICU ward at home. In this study, all mothers of newborns who are hospitalized in Amirkola and Ayatollah Rouhani Babol pediatric hospitals in NICU during 2021, are considered as the study population and enter the study if satisfied.

**Participants/Inclusion and exclusion criteria**

Inclusion criteria: be literate; no history of mental illness in the mother; 7 days have passed since the baby was admitted; score of 43 or higher on the General Sleep Disorder Scale; lack of sensitivity to medicinal plants and food; no pregnancy complications such as diabetes and eclampsia and heart problems; no previous history of premature infants or hospitalized infants Exclusion criteria: infant hospitalization less than 14 days in the neonatal intensive care unit; the mother's unwillingness to continue participating in the study; occurrence of unpleasant events for the mother; maternal sensitivity to orange spring sweat.

**Intervention groups**

Intervention group A will consume Citrus Aurantium distillate syrup and group B will be the control group who will be given syrup prepared from water and sugar.

**Main outcome variables**

Mothers' anxiety; maternal sleep quality

•**Project description:**

− Rationale

Mothers of neonates admitted to the intensive care unit are prone to sleep disorders due to stress and anxiety. Some herbs have anti-anxiety and sedative properties. According to a report from the World Health Organization, the use of herbal medicines is on the rise in most countries due to their efficacy, lower risk and cost, and greater availability [1]. Bitter orange (Citrus aurantium) blossoms are one of the most widely used native medicinal plants in Iran. This plant is recognized as a sedative, soporific agent, appetizer, and reliever of heart palpitations. According to research, bitter orange blossoms contain compounds such as linalool, linalyl acetate, limonene, coumarin, and flavonoids. The contents of these compounds are greater in the blossoms than in the leaves [2]. Few studies have been conducted in humans on the effects of bitter orange blossom distillate (BOBD) in the postpartum period, and most of them have been aromatherapy or laboratory studies.

There is a high incidence of postpartum anxiety and sleep disorders among mothers with hospitalized infants [3], which can affect both mothers and infants. In addition, many mothers believe that herbal products are superior to chemical medications and prefer using them. Compounds found in the bitter orange are known to have soothing effects. In light of the aforementioned considerations and due to the lack of research in this area, this study was done.

**− Objectives**

The aim of this study was to investigate the effect of bitter orange blossom distillate on anxiety and sleep disorder in mothers with infants admitted to neonatal intensive care unit.

**− Methodology**

This randomized controlled clinical trial was conducted in 2021on 60 mothers with NICU-admitted infants in one of the medical teaching centers affiliated to Babol University of Medical Sciences, Iran. Permuted block randomization was used to assign the mothers to the experimental (bitter orange blossom distillate syrup) and control groups (syrup prepared from water and sugar).Given the impossibility of blinding the participants, allocation concealment was used to prevent the researcher from predicting the future allocation of the samples to the groups. In addition, the statistical consultant received coded data pertaining to the type of intervention in each group. (While blinding requires similarity, it was not possible to comply with this principle in the case of Bitter Orange Blossom Distillate because its smell has therapeutic effect which is part of the study. We didn't add this smell to the control group. The smell of syrup was different in the two groups, therefore, the patients were not blinded, but the shape, color, and consistency were the same. The study was conducted during the covid-19 pandemic, and there was no possibility of contamination between mothers, as mothers consumed syrup at home). A demographic questionnaire, the Spielberger State and Trait Anxiety Inventory (STAI) and the General Sleep Disorder Scale were employed to collect data. We assessed the level of anxiety and sleep disorder (in both groups before the intervention and again after the last day of the intervention).

The eligible participants signed written consent form to enter the research. A demographic questionnaire, the Spielberger State and Trait Anxiety Inventory (STAI) and the GSDS were employed to collect data. Numerous studies have investigated and confirmed the validity and reliability of the STAI [4, 5].

The participants in the experimental group (n=30) took 100 mL syrup prepared by the researcher (under the supervision of an expert group in Iranian traditional medicine) three times daily at 8 AM, 2 PM and 8 PM for seven days. (The syrup was prepared as follows: The researcher gradually dissolved sugar (66.7 g) in 100 mL BOBD until a uniform solution was obtained (sugar to distillate ratio is based on the USP^[[1]](#footnote-1)^ standard.). Afterward, 30 mL of the resulting solution was further diluted with 70 mL water to raise the volume to 100 mL).

In the control group (n=30), the participants were given 100 mL syrup with the same percentage of the sweetness of bitter orange syrup three times daily at the same times as the experimental group for seven days. The researcher conducted daily telephone follow-ups to ensure that all the participants in the groups consumed the prepared solutions.

− **Data management and analysis**

The collected data were analyzed in SPSS Version 22. Descriptive statistics were presented as mean (± standard deviation (SD)), frequency, and percentage. To assess the effect of intervention on primary outcomes we used analysis of covariance, after verifying and confirming the assumptions of ANCOVA—specifically, the normality within subgroups and the absence of significant interaction between group and covariate—the p-value for the group effect was reported. The level of significance was set at P <0.05. In all tests, a two-tailed statistical test was utilized.

**• Ethical considerations**

Ethics Committee of Babol University of Medical Sciences

IR.MUBABOL.REC.2019.393

The eligible participants signed written consent form to enter the research

**• Gender issues**

Female (Mothers participated in this research) has above 18 years old

**The inclusion criteria** comprised being literate, not having a history of neurological and mental illnesses, the infants being in the NICU for seven days, the mothers scoring 43 or higher on the GSDS, not having allergies to medicinal and food plants, not having a history of diabetes, not having had a premature or NICU-admitted infant before, and not having pregnancy complications such as diabetes, eclampsia and heart problems.

**The exclusion criteria** were the infant's hospitalization in the NICU for fewer than 14 days, the mother's refusal to continue participation in the study, occurrence of unpleasant events for the mother, the mother's sensitivity to BOBD, and the mother's need for sleep disorder or anxiety medications during the intervention

• References

[1] Kamalifard M, Khalili AF, Namadian M, Herizchi S, Ranjbar Y. Comparison of the effect of lavender and bitter orange on depression in menopausal women: a triple-blind randomized controlled trial. Int J Womens Health Reprod Sci. 2017;5(3):224-30. doi:[10.15296/ijwhr.2017.40](http://dx.doi.org/10.15296/ijwhr.2017.40)

[2] Abbasnejad, M, Keramat B, Esmaili Mahani, S, Rezaeezade-Roukerd M. Effect of Hydro-Methanolic Extract of Sour Orange Flowers, Citrus Aurantium, on Pentylentetrazole Induced Seizure in Male Rats. J Babol Univ Med Sci 2012; 14 (5) :20-28. URL: http://jbums.org/article-1-4174-en.html

[3] Abdullah KL, Chong MC, Chua YP, Al Kawafha MM. Stress, anxiety, depression and sleep disturbance among Jordanian mothers and fathers of infants admitted to neonatal intensive care unit: A preliminary study.

J Pediatr Nurs. 2017 Sep-Oct;36:132-40. doi: 10.1016/j.pedn.2017.06.007. Epub 2017 Jun 19.

[4] Gholami Booreng F, Mahram B, Kareshki H. Construction and Validation of a Scale of Research Anxiety for Students. Iranian Journal of Psychiatry and Clinical Psychology. 2017;23(1):78-93.doi: 10.18869/nirp.ijpcp.23.1.78

[5] Adeli M, Alirezaei S. Comparison of short abdominal skin to skin and Kangaroo contact after birth on state and trait anxiety. Journal of Torbat Heydariyeh University of Medical Sciences (jms). 2017; 5 (2) :1-10 URL: <http://jms.thums.ac.ir/article-1-405-en.html>

1. United States Pharmacopeia # 1 [↑](#footnote-ref-1)
